# Value of chemotherapy post immunotherapy in stage IV non-small cell lung cancer (NSCLC)

**DOI:** 10.18632/oncotarget.28444

**Published:** 2023-05-26

**Authors:** Hazem I. Assi, Maroun Bou Zerdan, Mohammad Hodroj, Makram Khoury, Nour Sabiha Naji, Ghid Amhaz, Reine Abou Zeidane, Fadi El Karak

**Affiliations:** ^1^Department of Internal Medicine, Division of Hematology and Oncology, Naef K. Basile Cancer Institute, American University of Beirut Medical Center, Beirut, Lebanon; ^2^Department of Internal Medicine, Division of Hematology and Oncology, Hotel Dieu de France University Hospital, Beirut, Lebanon; ^*^These authors contributed equally to this work

**Keywords:** chemotherapy, immunotherapy, non-small cell lung cancer

## Abstract

Background: Lung cancer is the number one cause of mortality among all types of cancer worldwide. Its treatment landscape has shifted from the classic chemotherapy alone to newer regimens based on the discovery of new immunotherapy and targeted therapy drugs. However, chemotherapy is still an option for treatment of advanced non-small cell lung cancer (NSCLC) after progression on immunotherapy alone or in combination with first-line chemotherapy.

Methods: This is a retrospective study based on chart review of patients diagnosed with advanced NSCLC cases who received Docetaxel as second or third line after being treated by immunotherapy and/or chemotherapy in previous lines. The data was collected from the medical records of physicians’ clinics in three different hospital centers in Lebanon over the period of 5 years from July 2015 until December 2020. February 2021 was data analysis cut off time. The main aim was to assess the role of Docetaxel post-chemoimmunotherapy for patients with diagnosed NSCLC.

Results: A total of 21 patients were included in this study. The majority of our patients were males (81%). As for histologic type, most patients had non-squamous lung cancer (67%) as compared to 33% who had squamous lung cancer. Overall, our study reported a 24% response rate to Docetaxel including stable disease and partial response and a median progression free survival (PFS) of 3 months. The mean time interval elapsed from diagnosis to the initiation of Docetaxel was 11.5 months.

Conclusion: New therapeutic options should be validated for the treatment of NSCLC in the second and subsequent lines of therapy considering the poor prognosis of this disease. The chemotherapy in second and third line may keep an important role in the treatment after progression on newer agents, but it needs more evidence in prospective studies including a larger number of patients.

## INTRODUCTION

Lung cancer is still the most common cause of worldwide cancer deaths for both men and women with a yearly estimated 1.6 million deaths. Its death rate exceeds that of the three common cancers: breast, colon and pancreatic combined together [1]. It is frequently diagnosed at an advanced stage and 5-year survival rates do not exceed 5% [2]. Around 85% of patients belong to a group labeled as non-small cell lung cancer (NSCLC), that is divided into several subtypes based on histological criteria, of which lung adenocarcinoma (LUAD) and lung squamous cell carcinoma (LUSC) subtypes constitute the majority [3]. Smoking is well known for its strong contribution in the development of lung cancer especially when it is related to small cell lung carcinoma (SCLC) and LUSC compared to LUAD which represents the most common histology in non-smoker patients. In addition, lung cancer in non-smokers has been associated with secondhand smoking, pollution and occupational carcinogens [4]. Lung cancer is a complex and heterogeneous disease at both molecular and genetic levels. Its treatment has changed with new discoveries from a physician’s preference of random protocols to an individualized plan according to the genetic alterations of the tumor. One possible route is to unravel the status of programmed death ligand-1 (PDL-1) in order to utilize immunotherapies [5].

In general, the treatment of lung cancer relied on conventional chemotherapies until late 2000s, when biological agents, currently known as targeted therapy, emerged as therapeutic options for NSCLC in case the tumors exhibit a driver mutation [6]. The number of targeted mutations extended over the past years to include epidermal growth factor receptor (EGFR), ROS1, BRAF, NTRK, RET, MET, RAS among others, and the different drugs many which target these mutations have been approved by the Food and Drug Administration (FDA) [7]. Check point inhibitors (CPIs) is another family of drugs that focused on targeting either PD-L1 (Atezolizumab, Durvalumab, Avelumab) or its receptor PD1 (Pembrolizumab, Nivolumab) [8]. These CPIs appeared with Nivolumab, being the first immunotherapy (IO) agent to be approved in second line treatment of NSCLC in 2015 based on the results of 2 phase III clinical trials. Moreover, Pembrolizumab (an anti-PD-1 antibody) and Atezolizumab (an anti-PD-L1 antibody) were included later for the second-line treatment and then studied in the frontline therapy due to their overall survival (OS) improvement and better toxicity profile [9]. In addition, Pembrolizumab was the first IO agent to be approved as monotherapy of advanced NSCLC with PD-L1 expression ≥50% and recently with PD-L1 expression ≥1% [10]. Even in the absence of PD-L1 expression, the combination of Pembrolizumab with selected combined chemotherapy (CT) showed OS advantage over that of CT alone in metastatic NSCLC [11].

In light of the new advancements in therapeutic options to treat NSCLC, we decided to conduct a retrospective study to report the use of Docetaxel in second or third-line treatment after chemo-immunotherapy in previous lines in patients diagnosed with advanced NSCLC.

## RESULTS

We included a total of 21 patients who have received Docetaxel as a treatment of non-small cell lung cancer to evaluate the response to this treatment in second or third line after chemo-immunotherapy. The patients were collected from the medical records of physicians’ clinics in three hospitals in the country.

Most of our patient population were males (81%) According to the histopathologic type, the nonsquamous non-small cell lung cancer was more frequent than the squamous cancer with a frequency of 67% (*N* = 14). Prior to the use of Docetaxel, the previous treatments consisted of the association of chemotherapy and immunotherapy as first-line treatment or chemotherapy alone followed by the addition of immunotherapy in the second line. Therefore, Docetaxel was used in the second line setting in 28.6% of the cases (*N* = 6) whereas it was used as a third-line treatment in 71.4% of the cases (*N* = 15). The used chemotherapeutical agents were Cisplatin, Carboplatin, Vinorelbine, Pemetrexed and Gemcitabine. However, the immunological agents used were, by order of frequency, Pembrolizumab (*N* = 12), Nivolumab (*N* = 6), Atezolizumab (*N* = 2) and Durvalumab (*N* = 1) in approximately 57%, 28.5%, 9.5% and 5% of the cases respectively. Table 1 shows the patients’ characteristics.

**Table 1:** Patients’ characteristics

Variable		*N* (%)
Gender	Males	17 (81%)
	Females	4 (19%)
Type of lung cancer	Non-squamous	14 (67%)
	Squamous	7 (33%)
Immunological agents (1st line)	Pembrolizumab	12 (57%)
	Nivolumab	6 (28.5%)
	Atezolizumab	2 (9.5%)
	Durvalumab	1 (5%)
Chemotherapeutic agents (1st line)	Cisplatin and Vinorelbine	3 (14.3%)
	Cisplatin and Pemetrexed	3 (14.3%)
	Carboplatin and Pemetrexed	11 (52.4%)
	Carboplatin and Gemcitabine	4 (19%)
Docetaxel	2nd line	6 (28.6%)
	3rd line	15 (71.4%)

**Figure 1:**
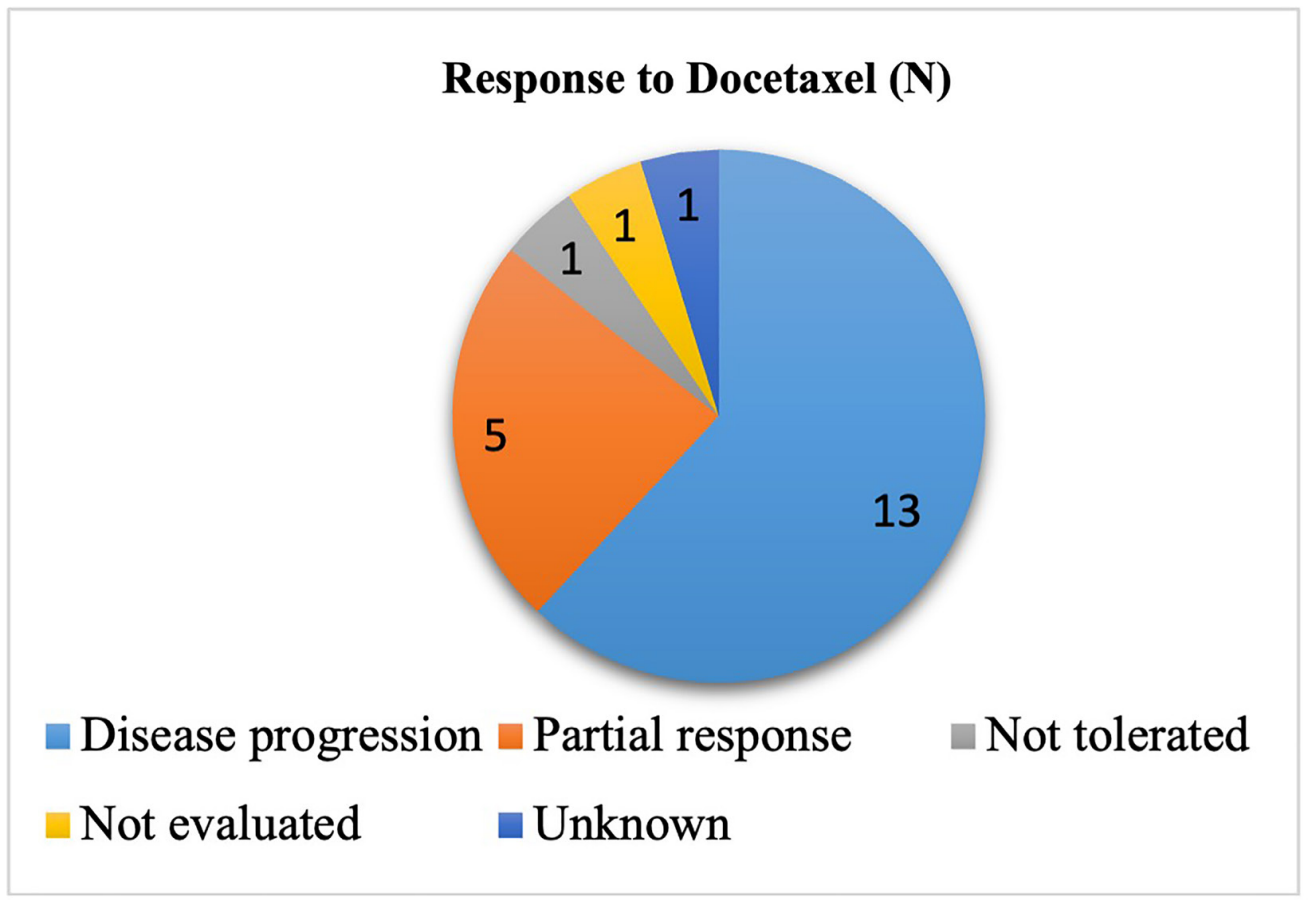
Response to docetaxel in second or third line treatment for non-small cell lung cancer after a treatment with chemo-immunotherapy.

The RECIST 1.1 criteria were used to assess response to Docetaxel. The results are reported In Figure 1.

Among the patients receiving Docetaxel, this treatment was not tolerated and then stopped in one patient after the first cycle. Disease progression was observed in 62% of the cases; however, a partial response/stable disease was obtained in almost 24% of the cases. The median PFS was 3 months.

We estimated the time interval elapsing between the date of diagnosis and the date of initiation of Docetaxel. The mean duration was estimated to 11.5 months with a slightly lower duration in the sub-group of patients experiencing disease progression, which was estimated to 10 months.

## DISCUSSION

Our results underline the dismal survival of lung cancer, particularly the non-small cell lung cancer, especially as the lines of treatment progress. This is concluded from the rate of disease progression and the time interval necessary for the initiation of Docetaxel. On the other hand, the comparison of our results with the previously exposed studies shows that the median PFS concluded in this study is almost similar to the mPFS reported in other studies for Docetaxel as second and third-line treatment [12–15] Docetaxel has been associated with increased response rate and higher one year survival when used alone as second-line therapy as seen in TAX 320 and TAX 317 clinical trials [12, 16]. Furthermore, a metanalysis published by Nagaraju et al. was comprised of 18 randomized controlled trials which included docetaxel in the standard treatment arm while kinase inhibitors, antineoplastic agents and monoclonal antibodies as the experimental arm for the treatment of stage III-TV NSCLC [17]. Overall, 9738 patients were included, and OS was better in patients who had been treated with Docetaxel as second-line for advanced NSCLC than in patients who received antineoplastic agents, kinase inhibitors and monoclonal antibodies [17].

Interestingly, Docetaxel is the first FDA approved agent to be used as second-line treatment of advanced NSCLC [18]. However, we should shed light on the toxicity profile of Docetaxel as it can cause myelosuppression in over half of the patients treated on an every-3-weeks schedule [18]. A study conducted by Wang et al. revealed that the frequency of administration of Docetaxel was related to the occurrence of neutropenia as the incidence rate of neutropenia after once-per-cycle was significantly higher than that observed after twice-per cycle. [19] As well, the duration of one cycle also plays a role since the incidence was significantly higher in 21-day cycles than in 28-day cycles [19]. Other than myelosuppression, it can lead to neuropathy, diarrhea, stomatitis, nausea and vomiting. Since the latter adverse effects were schedule-dependent, weekly dosing was initiated to limit toxicity. In fact, the weekly administration of Docetaxel was shown to have a better toxicity profile with similar efficacy when compared to 3-weekly administration [18]. Therefore, Docetaxel with controlled side effects may serve as an essential second and third-line agent after chemoimmunotherapy.

### The role of chemotherapy with/without targeted therapy in second-line treatment post failure of immunotherapy

Although CPIs have been shown to improve OS, resistance to such agents is inevitable in some cases. Primary resistance to CPIs occurs when patients do not respond to CPIs as initial therapy; however, acquired resistance occurs when patients respond to CPIs but later relapse. Primary resistance is mainly due to the inhibition of anti-tumor immune responses. The latter happens due to tumor cell-intrinsic factors and extrinsic factors. The intrinsic factors include lack of tumor antigen expression, abnormal mitogen-activated protein kinase (MAPK) signaling, absence of PTEN expression, abnormal interferon gamma signaling and aberrant WNT/beta catenin signaling, among others [20]. Nevertheless, extrinsic factors essentially involve the activity of regulatory T cells, macrophages and myeloid suppressor cells [20]. For acquired resistance, several mechanisms, such as escape mutations in the tumor cell, absence of T cell function and loss of T cell recognition due to tumor antigen presentation downregulation, lead to this resistance [20]. Moreover, NSCLC patients whose tumors lack a driver mutation and were formerly treated with IO in first line, either as monotherapy or in combination with CT, have very limited treatment options after the progression of their disease. As IO moved up to the first-line treatment, patients face the obstacle of shifting from single-agent IO or chemoimmunotherapy combination to conventional systemic CT regimens with/without biological agents which exhibit higher toxicities and less efficacy compared to the first-line [21]. CT, when feasible, remains as the only viable option post progression on IO in the absence of targeted mutations, which directed lately the attention towards evaluating the efficacy of salvage CT after IO (SCAI) [22]. Data from several cohorts supported an emerging hypothesis of a promising role for IO in increasing the sensitivity of the tumor to the subsequent CT [23].

The use of a single-agent CT as second line resulted in poor clinical outcomes in clinical trials with median progression free survival (mPFS) ranging between 2.6 and 2.9 months and median overall survival (mOS) of 7 to 8.3 months with the use of docetaxel [24]. Of importance, a phase III trial reported that Pemetrexed is equivalent to Docetaxel in second-line treatment of advanced NSCLC [13]. In addition, the combination strategy of Pemetrexed and Carboplatin did not improve the survival outcomes compared to Pemetrexed monotherapy in second line [25]. Only few drugs were approved by FDA to be used in the second-line treatment based on histology, if not previously used in the first-line settings, such as Gemcitabine, Pemetrexed for non-squamous NSCLC, Docetaxel ± Ramucirumab for all histologic types, and Afatinib for squamous cell histology only [26]. For example, a phase III clinical trial by Garon et al. reported that the combination of Docetaxel + Ramucirumab as second-line therapy improved response, PFS and OS in patients with stage IV NSCLC [27]. Essentially, the combination of Docetaxel + Nintedanib was used in Europe and Latin America in second-line treatment of non-squamous NSCLC in 2014. The combination of Nintedanib, a triple angiokinase inhibitor, with Docetaxel was studied in LUME-Lung 1, a phase III trial that involved patients who had stage IIIB/IV recurrent NSCLC of all histologies. This study revealed a significant improvement in PFS for both adenocarcinoma and squamous NSCLC in the combination arm over Docetaxel/Placebo, and it showed improvement of OS only in adenocarcinoma NSCLC patients [28]. Recently, results from VARGADO study which addressed the use of Docetaxel/Nintedanib as third-line therapy after failing CT and CPI, were published showing improvement of PFS. In this ongoing German prospective study, cohort C included patients receiving Docetaxel/Nintedanib as second-line therapy after they have failed chemoimmunotherapy as frontline treatment. The authors of the study concluded that Docetaxel/Nintedanib may have a potential clinical benefit in patients who failed first-line IO [29].

Few months ago, Bersanelli et al. published the results of a large retrospective multicenter study that involved patients with advanced NSCLC from 20 Italian centers between 2013 and 2019 to assess the benefit of CT in second line and beyond after progressing on IO. The study population included 342 patients, 86 (25.1%) of which received salvage CT post IO as second line. Despite the lack of statistical significance, an increased benefit was observed in this patient population. The study group showed that the line of treatment is one of the major determinants of survival in the settings of post IO treatment and that the use of platinum-based doublets as a second line achieved the highest overall response rate (ORR). As well, a prognostic score for the use of CT post IO failure was established, stratifying the patients into groups of good, intermediate and poor prognosis as this can help clinicians in tailoring the best treatment for each group [30]. Furthermore, Table 2 shows several important clinical trials that studied available second-line therapies for NSCLC patients previously treated with immunotherapy.

**Table 2:** List of clinical trials that studied available second-line therapies for NSCLC patients previously treated with immunotherapy

Clinical trial sponsor	NSCLC type	Therapeutic agent	Response	PFS (months)	OS (months)
Bersanelli et al. [30]	All histologies	Platinum combination or Gemcitabine alone or Taxane-based or Vinorelbine alone or other	22.8%	4.1 (95% CI 3.4–4.8)	6.8 (95% CI 5.5–8.1)
Grohé et al. [29] (VARGADO study)	Adenocarcinoma	Docetaxel + Nintedanib	58% PR, 25% SD, 83% DCR	5.5 (95% CI, 1.9–8.7)	Not reported
Garon et al. [27]	All histologies	Docetaxel + Ramucirumab vs. Docetaxel + placebo	23% vs. 14% (*p* < 0.001)	4.5 vs. 3.0 (*p* < 0.0001)	10.5 vs. 9.1 (*p* = 0.023)
Reck et al. [28]	Adenocarcinoma	Docetaxel + Nintedanib vs. Docetaxel + placebo	DCR 54.0% vs. 43.0% (*p* < 0.0001)	3.4 vs. 2.7 (*p* = 0.0019)	12.6 vs. 10.3 (*p* = 0.0359)
Hanna et al. [13]	All histologies	Docetaxel vs. Pemetrexed	9.1% vs. 8.8% (*p* = 0.105)	2.9 for each	8.3 vs. 7.9 (*p* = 0.226)
Crinò et al. [36]	All histologies	Gemcitabine	19% PR, 31% SD	Not reported	Not reported

Abbreviations: CI: Confidence interval; DCR: Disease control rate; OS: Overall survival; PFS: Progression free survival; PR: Partial response, SD: Stable Disease.

### The role of chemotherapy with/without targeted therapy in third-line treatment post failure of first-line chemotherapy and second-line immunotherapy

First-line and second-line CT have been demonstrated, since decades, to be effective in the treatment of patients with inoperable advanced NSCLC. However, the initiation of a third-line treatment was always debatable without clear evidence to support. The decision of offering a third-line CT treatment in NSCLC was always linked to the assessment of the performance status (PS) of the patients to identify the eligible candidates [31]. A retrospective study indicated a longer survival after third-line CT when used in patients with low PS compared to high PS and suggested a possible association with increased OS [32]. Other than PS, several important prognostic factors, which are reported for the initiation of first-line chemotherapy, also come into play for third-line therapy; these include age <70, absence of cancer related symptoms and weight loss, absence of extrathoracic metastases and short smoking history [31]. In addition, disease control after the first two lines is an essential factor to consider when initiating a third line treatment. Importantly, a study by Girard et al. revealed that disease control after first- and second-line treatments was highly associated with a greater overall survival after third-line treatment. Median survival was 10.3 months in case of disease control after the first two lines while it was 4.0 months in case of progression [31]. To note, the third-line regimens used in the latter study included Docetaxel, Pemetrexed, Gemcitabine, Vinorelbine and Paclitaxel among others [31].

Nevertheless, over the past decade, the field of NSCLC treatment underwent major changes with the emergence of IO as a cornerstone of the current treatment. However, most of the NSCLC patients treated with IO as second-line experience disease progression with a small group having exceptionally long-lasting responses and survival [33]. Therefore, establishing effective third-line therapies after first-line platinum-based CT and second-line IO is considered crucial for a large number of patients. Since some antiangiogenic agents such as Nintedanib and Ramucirumab lead to significant increase in OS when combined to CT in second line after failure of platinum-based combination CT, these agents might represent reasonable candidates for combinations with CT in third line treatment [34]. This topic was heavily studied lately with many retrospective cohorts assessing the results of different agents. A systemic review of real-world observational studies reported mOS range of 2.8 to 12 months with CT in third-line treatment of advanced NSCLC [14]. Moreover, Schvartsman et al. conducted a retrospective study at MD Anderson Cancer Center showing that ORR was higher in single-agent CT, in third-line and beyond, after the exposure to PDL-1 inhibitors compared to that from historical data in the pre-anti-PDL-1 era [22]. Recently, another study investigated the efficacy of CT after PDL-1 inhibitor vs CT alone in 1439 advanced NSCLC individuals. The results highlighted that CT post PDL-1 inhibitors in third line achieved a higher ORR of 18.9% compared to 11% in patients who received CT in second line with the lack of previous exposure to anti PD-1 agents despite the disproportion between the line settings of the two arms [35]. Of importance, the efficacy of Docetaxel/Nintedanib combination in third-line treatment of advanced lung adenocarcinoma post failure of platinum-based CT followed by IO was first reported in a small retrospective study where 11 out of 390 patients from 108 Spanish centers met the inclusion criteria. The mPFS of the combination in third-line was 3.2 months, reinforcing the importance of an optimal therapeutic sequence in the treatment of NSCLC. Furthermore, in the prospective and non-interventional study VARGADO, the mPFS was 5.5 months with an ORR of 58% for 22 patients who received Docetaxel/Nintedanib as third-line treatment after failing CT and IO. However, the low number of patients represents a major and clear limitation of the study. The primary endpoint of this cohort is OS which is still immature and not available at the time of this publication [21].

Even though third- line treatments sometimes pose a survival benefit, their main aim should emphasize on symptom palliation and PS control, which was interestingly achieved in more than 90% of the patients in Girard et al.’ s retrospective study [31]. Meanwhile, the debate of initiating a third- line treatment is still ongoing with more evidence supporting its use in selected cases.

## MATERIALS AND METHODS

### Study design and patient selection

This is a retrospective cohort study performed based on chart review of patients from physicians’ clinic across three different hospital centers in Lebanon over a period of 5 years from July 2015 until December 2020. February 2021 was data analysis cut off time. All medical charts were reviewed to obtain demographic data, clinical data, pathologic findings, site of metastases as well as therapeutic modalities (chemo/immunotherapy) used in 1st and/or 2nd lines followed by Docetaxel use.

### Data analysis

All collected data were entered and statistical analysis was performed using IBM SPSS v.25 with the use of identification numbers. Patient identifiers like names or case numbers were concealed throughout the whole study. Data analysis included baseline descriptive statistical analysis for demographics, patient characteristics, histologic type of cancer, treatment characteristics as well as survival analysis based on the time of last clinical follow up in the charts and the time of revision cutoff, which was February 2021.

## CONCLUSIONS

NSCLC’s treatment field has experienced vital advancements with use of IO, which moved up to become a frontline treatment in a short period of time. By increasing survival, IO is changing the future treatment perspective. Despite the promising results of IO, a majority of patients are still experiencing progression of their disease urging the establishment of new treatment options. Data from several studies suggest a promising role for CT with/without targeted agents in the second-line treatment post IO. Importantly, Docetaxel has been intensely investigated in multiple clinical trials and has shown proven efficacy as a second or third-line agent post-chemoimmunotherapy in patients with advanced NSCLC. Docetaxel will remain a main therapeutic agent in the treatment of NSCLC with more studies emphasizing on its benefit. This is due to the fact that most of the available data are based on retrospective cohorts and need to be supported by prospective studies to gain valuable evidence. In addition, an important role for CT with/without targeted agents as a third line after IO failure is suggested by different studies. These studies are still limited by the small population and the retrospective settings. This role might be validated in the presence of many preliminary evidence that support the hypothesis of increased tumor sensitivity to CT post exposure to IO agents. Therefore, large multicenter prospective randomized trials are needed to provide the clinical evidence for the use of CT in second line and third-line post IO failure.

## References

[1] Siegel R , Ma J , Zou Z , Jemal A . Cancer statistics, 2014. CA Cancer J Clin. 2014; 64:9–29. 10.3322/caac.21208. 24399786

[2] Barlesi F , Dixmier A , Debieuvre D , Raspaud C , Auliac JB , Benoit N , Bombaron P , Moro-Sibilot D , Audigier-Valette C , Asselain B , Egenod T , Rabeau A , Fayette J , et al. Effectiveness and safety of nivolumab in the treatment of lung cancer patients in France: preliminary results from the real-world EVIDENS study. Oncoimmunology. 2020; 9:1744898. 10.1080/2162402X.2020.1744898. 33457089 PMC7790497

[3] Herbst RS , Morgensztern D , Boshoff C . The biology and management of non-small cell lung cancer. Nature. 2018; 553:446–54. 10.1038/nature25183. 29364287

[4] Sun S , Schiller JH , Gazdar AF . Lung cancer in never smokers--a different disease. Nat Rev Cancer. 2007; 7:778–90. 10.1038/nrc2190. 17882278

[5] Zhang J , Fujimoto J , Zhang J , Wedge DC , Song X , Zhang J , Seth S , Chow CW , Cao Y , Gumbs C , Gold KA , Kalhor N , Little L , et al. Intratumor heterogeneity in localized lung adenocarcinomas delineated by multiregion sequencing. Science. 2014; 346:256–59. 10.1126/science.1256930. 25301631 PMC4354858

[6] Mok TS , Wu YL , Thongprasert S , Yang CH , Chu DT , Saijo N , Sunpaweravong P , Han B , Margono B , Ichinose Y , Nishiwaki Y , Ohe Y , Yang JJ , et al. Gefitinib or carboplatin-paclitaxel in pulmonary adenocarcinoma. N Engl J Med. 2009; 361:947–57. 10.1056/NEJMoa0810699. 19692680

[7] Subbiah V , Gainor JF , Rahal R , Brubaker JD , Kim JL , Maynard M , Hu W , Cao Q , Sheets MP , Wilson D , Wilson KJ , DiPietro L , Fleming P , et al. Precision Targeted Therapy with BLU-667 for *RET*-Driven Cancers. Cancer Discov. 2018; 8:836–49. 10.1158/2159-8290.CD-18-0338. 29657135

[8] Imyanitov EN , Iyevleva AG , Levchenko EV . Molecular testing and targeted therapy for non-small cell lung cancer: Current status and perspectives. Crit Rev Oncol Hematol. 2021; 157:103194. 10.1016/j.critrevonc.2020.103194. 33316418

[9] Herbst RS , Baas P , Kim DW , Felip E , Pérez-Gracia JL , Han JY , Molina J , Kim JH , Arvis CD , Ahn MJ , Majem M , Fidler MJ , de Castro G Jr , et al. Pembrolizumab versus docetaxel for previously treated, PD-L1-positive, advanced non-small-cell lung cancer (KEYNOTE-010): a randomised controlled trial. Lancet. 2016; 387:1540–50. 10.1016/S0140-6736(15)01281-7. 26712084

[10] Mok TSK , Wu YL , Kudaba I , Kowalski DM , Cho BC , Turna HZ , Castro G Jr , Srimuninnimit V , Laktionov KK , Bondarenko I , Kubota K , Lubiniecki GM , Zhang J , et al. Pembrolizumab versus chemotherapy for previously untreated, PD-L1-expressing, locally advanced or metastatic non-small-cell lung cancer (KEYNOTE-042): a randomised, open-label, controlled, phase 3 trial. Lancet. 2019; 393:1819–30. 10.1016/S0140-6736(18)32409-7. 30955977

[11] Gandhi L , Rodríguez-Abreu D , Gadgeel S , Esteban E , Felip E , De Angelis F , Domine M , Clingan P , Hochmair MJ , Powell SF , Cheng SY , Bischoff HG , Peled N , et al. Pembrolizumab plus Chemotherapy in Metastatic Non-Small-Cell Lung Cancer. N Engl J Med. 2018; 378:2078–92. 10.1056/NEJMoa1801005. 29658856

[12] Shepherd FA . Second-line chemotherapy for non-small cell lung cancer. Expert Rev Anticancer Ther. 2003; 3:435–42. 10.1586/14737140.3.4.435. 12934656

[13] Hanna N , Shepherd FA , Fossella FV , Pereira JR , De Marinis F , von Pawel J , Gatzemeier U , Tsao TC , Pless M , Muller T , Lim HL , Desch C , Szondy K , et al. Randomized phase III trial of pemetrexed versus docetaxel in patients with non-small-cell lung cancer previously treated with chemotherapy. J Clin Oncol. 2004; 22:1589–97. 10.1200/JCO.2004.08.163. 15117980

[14] Davies J , Patel M , Gridelli C , de Marinis F , Waterkamp D , McCusker ME . Real-world treatment patterns for patients receiving second-line and third-line treatment for advanced non-small cell lung cancer: A systematic review of recently published studies. PLoS One. 2017; 12:e0175679. 10.1371/journal.pone.0175679. 28410405 PMC5391942

[15] Leger PD , Rothschild S , Castellanos E , Pillai RN , York SJ , Horn L . Response to salvage chemotherapy following exposure to immune checkpoint inhibitors in patients with non-small cell lung cancer. J Clin Oncol. 2017 (15_suppl); 35:9084. 10.1200/JCO.2017.35.15_suppl.9084.

[16] Fossella FV , DeVore R , Kerr RN , Crawford J , Natale RR , Dunphy F , Kalman L , Miller V , Lee JS , Moore M , Gandara D , Karp D , Vokes E , et al. Randomized phase III trial of docetaxel versus vinorelbine or ifosfamide in patients with advanced non-small-cell lung cancer previously treated with platinum-containing chemotherapy regimens. The TAX 320 Non-Small Cell Lung Cancer Study Group. J Clin Oncol. 2000; 18:2354–62. 10.1200/JCO.2000.18.12.2354. 10856094

[17] Nagaraju C , Vaidya G , Jain AS , Nair AP , Chandrappa S , Srinivasa C , Suresh KP , Patil SS , Shivananda B , Kollur SP , Shivamallu C . Overall Survival Prediction of Docetaxel-based Second-line Treatment for Advanced Non-small Cell Lung Cancer: A Systematic Review and Meta-analysis. Oman Med J. 2022; 37:e419. 10.5001/omj.2022.86. 36341003 PMC9618033

[18] Saloustros E , Georgoulias V . Docetaxel in the treatment of advanced non-small-cell lung cancer. Expert Rev Anticancer Ther. 2008; 8:1207–22. 10.1586/14737140.8.8.1207. 18699760

[19] Wang F , Zhao C , Wen X , Zheng Q , Li L . Factors affecting the efficacy and safety of docetaxel combined with platinum in the treatment of advanced non-small cell lung cancer. Expert Rev Clin Pharmacol. 2021; 14:1295–303. 10.1080/17512433.2021.1976638. 34488513

[20] Sharma P , Hu-Lieskovan S , Wargo JA , Ribas A . Primary, Adaptive, and Acquired Resistance to Cancer Immunotherapy. Cell. 2017; 168:707–23. 10.1016/j.cell.2017.01.017. 28187290 PMC5391692

[21] Santos ES . Treatment options after first-line immunotherapy in metastatic NSCLC. Expert Rev Anticancer Ther. 2020; 20:221–28. 10.1080/14737140.2020.1738930. 32141356

[22] Schvartsman G , Peng SA , Bis G , Lee JJ , Benveniste MFK , Zhang J , Roarty EB , Lacerda L , Swisher S , Heymach JV , Fossella FV , William WN . Response rates to single-agent chemotherapy after exposure to immune checkpoint inhibitors in advanced non-small cell lung cancer. Lung Cancer. 2017; 112:90–95. 10.1016/j.lungcan.2017.07.034. 29191606

[23] Park SE , Lee SH , Ahn JS , Ahn MJ , Park K , Sun JM . Increased Response Rates to Salvage Chemotherapy Administered after PD-1/PD-L1 Inhibitors in Patients with Non-Small Cell Lung Cancer. J Thorac Oncol. 2018; 13:106–11. 10.1016/j.jtho.2017.10.011. 29101058

[24] Shepherd FA , Dancey J , Ramlau R , Mattson K , Gralla R , O’Rourke M , Levitan N , Gressot L , Vincent M , Burkes R , Coughlin S , Kim Y , Berille J . Prospective randomized trial of docetaxel versus best supportive care in patients with non-small-cell lung cancer previously treated with platinum-based chemotherapy. J Clin Oncol. 2000; 18:2095–103. 10.1200/JCO.2000.18.10.2095. 10811675

[25] Ardizzoni A , Tiseo M , Boni L , Vincent AD , Passalacqua R , Buti S , Amoroso D , Camerini A , Labianca R , Genestreti G , Boni C , Ciuffreda L , Di Costanzo F , et al. Pemetrexed versus pemetrexed and carboplatin as second-line chemotherapy in advanced non-small-cell lung cancer: results of the GOIRC 02-2006 randomized phase II study and pooled analysis with the NVALT7 trial. J Clin Oncol. 2012; 30:4501–7. 10.1200/JCO.2012.43.6758. 23109689

[26] Soria JC , Felip E , Cobo M , Lu S , Syrigos K , Lee KH , Göker E , Georgoulias V , Li W , Isla D , Guclu SZ , Morabito A , Min YJ , et al. Afatinib versus erlotinib as second-line treatment of patients with advanced squamous cell carcinoma of the lung (LUX-Lung 8): an open-label randomised controlled phase 3 trial. Lancet Oncol. 2015; 16:897–907. 10.1016/S1470-2045(15)00006-6. 26156651

[27] Garon EB , Ciuleanu TE , Arrieta O , Prabhash K , Syrigos KN , Goksel T , Park K , Gorbunova V , Kowalyszyn RD , Pikiel J , Czyzewicz G , Orlov SV , Lewanski CR , et al. Ramucirumab plus docetaxel versus placebo plus docetaxel for second-line treatment of stage IV non-small-cell lung cancer after disease progression on platinum-based therapy (REVEL): a multicentre, double-blind, randomised phase 3 trial. Lancet. 2014; 384:665–73. 10.1016/S0140-6736(14)60845-X. 24933332

[28] Reck M , Kaiser R , Mellemgaard A , Douillard JY , Orlov S , Krzakowski M , von Pawel J , Gottfried M , Bondarenko I , Liao M , Gann CN , Barrueco J , Gaschler-Markefski B , et al. Docetaxel plus nintedanib versus docetaxel plus placebo in patients with previously treated non-small-cell lung cancer (LUME-Lung 1): a phase 3, double-blind, randomised controlled trial. Lancet Oncol. 2014; 15:143–55. 10.1016/S1470-2045(13)70586-2. 24411639

[29] Grohé C , Gleiber W , Haas S , Losem C , Mueller-Huesmann H , Schulze M , Franke C , Basara N , Atz J , Kaiser R . Nintedanib plus docetaxel after progression on immune checkpoint inhibitor therapy: insights from VARGADO, a prospective study in patients with lung adenocarcinoma. Future Oncol. 2019; 15:2699–706. 10.2217/fon-2019-0262. 31282758

[30] Bersanelli M , Buti S , Giannarelli D , Leonetti A , Cortellini A , Russo GL , Signorelli D , Toschi L , Milella M , Pilotto S , Bria E , Proto C , Marinello A , et al. Chemotherapy in non-small cell lung cancer patients after prior immunotherapy: The multicenter retrospective CLARITY study. Lung Cancer. 2020; 150:123–31. 10.1016/j.lungcan.2020.10.008. 33130353

[31] Girard N , Jacoulet P , Gainet M , Elleuch R , Pernet D , Depierre A , Dalphin JC , Westeel V . Third-line chemotherapy in advanced non-small cell lung cancer: identifying the candidates for routine practice. J Thorac Oncol. 2009; 4:1544–49. 10.1097/JTO.0b013e3181bbf223. 19884862

[32] Tatli AM , Arslan D , Uysal M , Goksu SS , Gunduz SG , Coskun HS , Ozdogan M , Savas B , Bozcuk HS . Retrospective analysis of third-line chemotherapy in advanced non-small cell lung cancer. J Cancer Res Ther. 2015; 11:805–9. 10.4103/0973-1482.146092. 26881522

[33] Gettinger S , Horn L , Jackman D , Spigel D , Antonia S , Hellmann M , Powderly J , Heist R , Sequist LV , Smith DC , Leming P , Geese WJ , Yoon D , et al. Five-Year Follow-Up of Nivolumab in Previously Treated Advanced Non-Small-Cell Lung Cancer: Results From the CA209-003 Study. J Clin Oncol. 2018; 36:1675–84. 10.1200/JCO.2017.77.0412. 29570421

[34] Brueckl WM , Reck M , Rittmeyer A , Kollmeier J , Wesseler C , Wiest GH , Christopoulos P , Tufman A , Hoffknecht P , Ulm B , Reich F , Ficker JH , Laack E . Efficacy of Docetaxel Plus Ramucirumab as Palliative Third-Line Therapy Following Second-Line Immune-Checkpoint-Inhibitor Treatment in Patients With Non-Small-Cell Lung Cancer Stage IV. Clin Med Insights Oncol. 2020; 14:1179554920951358. 10.1177/1179554920951358. 32884390 PMC7440727

[35] Kato R , Hayashi H , Chiba Y , Miyawaki E , Shimizu J , Ozaki T , Fujimoto D , Toyozawa R , Nakamura A , Kozuki T , Tanaka K , Teraoka S , Usui K , et al. Propensity score-weighted analysis of chemotherapy after PD-1 inhibitors versus chemotherapy alone in patients with non-small cell lung cancer (WJOG10217L). J Immunother Cancer. 2020; 8:e000350. 10.1136/jitc-2019-000350. 32066647 PMC7057433

[36] Crinò L , Mosconi AM , Scagliotti G , Selvaggi G , Novello S , Rinaldi M , Della Giulia M , Gridelli C , Rossi A , Calandri C , De Marinis F , Noseda M , Tonato M . Gemcitabine as second-line treatment for advanced non-small-cell lung cancer: A phase II trial. J Clin Oncol. 1999; 17:2081–85. 10.1200/JCO.1999.17.7.2081. 10561261

